# Visual Field Map Clusters in High-Order Visual Processing: Organization of V3A/V3B and a New Cloverleaf Cluster in the Posterior Superior Temporal Sulcus

**DOI:** 10.3389/fnint.2017.00004

**Published:** 2017-02-28

**Authors:** Brian Barton, Alyssa A. Brewer

**Affiliations:** ^1^Department of Cognitive Sciences, University of California, Irvine, IrvineCA, USA; ^2^Department of Linguistics, University of California, Irvine, IrvineCA, USA; ^3^Center for Hearing Research, University of California, Irvine, IrvineCA, USA

**Keywords:** V3A, V3B, posterior superior temporal sulcus, pSTS, cloverleaf clusters, visual field mapping, population receptive field modeling, visual cortex

## Abstract

The cortical hierarchy of the human visual system has been shown to be organized around retinal spatial coordinates throughout much of low- and mid-level visual processing. These regions contain visual field maps (VFMs) that each follows the organization of the retina, with neighboring aspects of the visual field processed in neighboring cortical locations. On a larger, macrostructural scale, groups of such sensory cortical field maps (CFMs) in both the visual and auditory systems are organized into roughly circular cloverleaf clusters. CFMs within clusters tend to share properties such as receptive field distribution, cortical magnification, and processing specialization. Here we use fMRI and population receptive field (pRF) modeling to investigate the extent of VFM and cluster organization with an examination of higher-level visual processing in temporal cortex and compare these measurements to mid-level visual processing in dorsal occipital cortex. In human temporal cortex, the posterior superior temporal sulcus (pSTS) has been implicated in various neuroimaging studies as subserving higher-order vision, including face processing, biological motion perception, and multimodal audiovisual integration. In human dorsal occipital cortex, the transverse occipital sulcus (TOS) contains the V3A/B cluster, which comprises two VFMs subserving mid-level motion perception and visuospatial attention. For the first time, we present the organization of VFMs in pSTS in a cloverleaf cluster. This pSTS cluster contains four VFMs bilaterally: pSTS-1:4. We characterize these pSTS VFMs as relatively small at ∼125 mm^2^ with relatively large pRF sizes of ∼2–8° of visual angle across the central 10° of the visual field. V3A and V3B are ∼230 mm^2^ in surface area, with pRF sizes here similarly ∼1–8° of visual angle across the same region. In addition, cortical magnification measurements show that a larger extent of the pSTS VFM surface areas are devoted to the peripheral visual field than those in the V3A/B cluster. Reliability measurements of VFMs in pSTS and V3A/B reveal that these cloverleaf clusters are remarkably consistent and functionally differentiable. Our findings add to the growing number of measurements of widespread sensory CFMs organized into cloverleaf clusters, indicating that CFMs and cloverleaf clusters may both be fundamental organizing principles in cortical sensory processing.

## Introduction

In many mammals, including humans, low and mid-level sensory cortex contains multiple, functionally specialized cortical field maps (CFMs), in which neurons whose sensory receptive fields are positioned next to one another in sensory feature space are located next to one another in cortex (for additional discussion, see Kaas J.H., 1997; Krubitzer, 2007; Wandell et al., 2007; Barton et al., 2012; Brewer and Barton, 2012b, 2016). The human visual system is organized around visuospatial coordinates throughout much of the cortical hierarchy. These regions contain visual field maps (VFMs) that each follows the organization of the retina, with neighboring aspects of the visual field processed in neighboring cortical locations.

It is critical to the investigation of the structure and function of human visual cortex to identify and characterize these VFMs. Each VFM performs a specific computation or set of computations that underlie particular perceptual behaviors, which typically become more complex as the neural processing continues up through the visual processing hierarchy (Sereno et al., 1995b; Shipp et al., 1995; Tootell et al., 1995; Engel et al., 1997; Van Essen, 2003). Each VFM thus facilitates the comparison and combination of the visual information carried by various specialized neuronal populations. Measuring the organization of individual VFMs helps differentiate the stages of distinct visual processing pathways and can be used to track how the cortex changes under various disorders (Baseler et al., 1999, 2002, 2011; Morland et al., 2001; Fine et al., 2003; Hoffmann et al., 2003, 2012, 2015; Van Essen, 2003; Chklovskii and Koulakov, 2004; Smirnakis et al., 2005; Wandell et al., 2005; Brewer, 2009; Muckli et al., 2009; Barton and Brewer, 2015). Furthermore, VFMs serve as excellent and reliable localizers for investigations of particular visual functions across individuals (Press et al., 2001; Huk et al., 2002; Brewer et al., 2005; Silver et al., 2005; Sereno and Amador, 2006; Wandell et al., 2007; Amano et al., 2009; Arcaro et al., 2009; Kolster et al., 2009, 2010; Brewer and Barton, 2014).

As greater numbers of VFMs have been defined in human visual cortex, a natural question to ask is whether there is a macrostructural organizing principle for the distribution of these maps across visual cortex (Hasson et al., 2002; Malach et al., 2002; Brewer et al., 2005; Tyler and Wade, 2005; Op de Beeck et al., 2008; Kolster et al., 2009). A basic approach with early VFMs has been to define strings of VFMs along contiguous strips of occipital cortex, with adjacent portions (boundaries) of the maps representing similar portions of space, but performing different computations. As additional VFMs have been defined across more extensive regions of visual cortex, more complex macrostructural organizing principles for visual cortex have been proposed (Kaas J., 1997; Hasson et al., 2002; Malach et al., 2002; Grill-Spector and Malach, 2004; Brewer et al., 2005; Tyler and Wade, 2005; Op de Beeck et al., 2008; Kolster et al., 2009; Brewer and Barton, 2012b). A growing body of evidence on the macrostructure of VFMs in human (Brewer et al., 2005; Wandell et al., 2005, 2007; Kolster et al., 2010; Brewer and Barton, 2012b) and macaque visual cortex (Kolster et al., 2009) has indicated that many, if not all, VFMs are organized into roughly circular cloverleaf clusters.

Visual field maps in a cloverleaf cluster are organized such that the central foveal representation of each VFM is positioned in the center of the cluster, with more peripheral representations of space represented in more peripheral positions in the cluster in a smooth, orderly fashion. The representation of any given polar angle of space for each VFM extends out from the center to the periphery of the cloverleaf cluster, effectively spanning the radius of the cluster like a spoke on a wheel. We describe this organization as being radially orthogonal. It is likely that this cluster organization, like the topographic organization of VFMs, allows for efficient connectivity among neurons that represent nearby aspects in visual space (Mitchison, 1991; Chklovskii and Koulakov, 2004; Shapley et al., 2007; Moradi and Heeger, 2009). The cloverleaf cluster organization may thus be important for minimizing the length of axons connecting sensory maps within and between clusters, allowing for a more efficient ratio of brain matter to skull capacity.

In addition, the spatial organization of VFMs may also play a role in coordinating neural computations. For example, the neurons within a cluster might share short-term information storage or mechanisms that coordinate neural timing (Brewer et al., 2005; Wandell et al., 2005). Similarly, it is likely that functional specializations for perception are organized around the activities within these clusters rather than single VFMs (Bartels and Zeki, 2000). This cluster-based computational organization has been demonstrated in the most detail in the homologous human (TO or hMT+) and macaque (MT+) clusters comprising the VFMs involved in motion processing, the MT cluster (Kolster et al., 2009, 2010). In macaque, the MT cluster has been show to contain four VFMs (MT, MST, FST, and V4t), all of which subserve unique stages in visual motion perception. The cluster organization is not thought to be driving the common functions, but rather reflects how multiple stages in a visual processing pathway might arise during development across individuals and during evolution across species (Krubitzer, 2007; Brewer and Barton, 2016).

The first complete cloverleaf cluster to be discovered lies just adjacent to the dorsal lower vertical meridian representation of human V3d and/or LO-1, although such cluster terminology was not yet in use (Brewer et al., 2005; Wandell et al., 2005). This cluster is composed of V3A and V3B (Smith et al., 1998, 2001; Press et al., 2001), two VFMs which share a discrete foveal representation within the transverse occipital sulcus (TOS) at the base of the intraparietal sulcus (IPS) (Wandell et al., 2007; Silver and Kastner, 2009). V3A has structural similarities to macaque V3A and is thought to play a role in human motion processing; the computations subserved by V3B and its homology to macaque are not yet known (Tootell et al., 1997, 1998; Smith et al., 1998; Press et al., 2001). Here, we present the first extensive characterization of the V3A/B cluster using functional MRI (fMRI) and population receptive field (pRF) modeling (Dumoulin and Wandell, 2008).

Compared to the TOS, the human posterior superior temporal sulcus (pSTS) is largely uncharted in terms of representations of visual space. Previous reports have implicated this region in such functions as face perception, biological motion processing, and audiovisual integration (Hoffman and Haxby, 2000; Grossman and Blake, 2002; Beauchamp et al., 2004). The variety of high-level visual processing functions attributed to the pSTS indicates that this region represents a high level in the visual hierarchy. As the pSTS also likely plays an important role in audiovisual integration, it may also subserve computations related to higher-level auditory processing. Presently, we report the first definition and characterization of four hemifield VFMs in the pSTS, which are organized into the pSTS cloverleaf cluster. These measurements are important not only for characterizing the new pSTS cluster itself, but also for investigating visuospatial organization that represents higher tiers of the cortical visual hierarchy.

We characterize the anatomical consistency (i.e., location and surface area), distribution of pRF centers and sizes, and reliability of the six VFMs from these two cloverleaf clusters. As expected from previous measurements of cloverleaf clusters, these measurements are very similar for VFMs within each cluster and differ between the clusters, adding weight to the growing evidence for cloverleaf clusters as an organizing principle of the human cortical visual system.

## Materials and Methods

### Subject Recruitment and Characterization

Five subjects (two females) participated in this study. Subjects were 24–36 years old, right-handed, had normal or corrected-to-normal visual acuity, and were expert at fixating on a central mark while attending to a moving stimulus during visual field mapping experiments. This study was carried out in accordance with the recommendations of the Institutional Review Board (Social and Behavioral Sciences Panel C) at the University of California, Irvine (UCI). All subjects gave written informed consent in accordance with the Declaration of Helsinki prior to the initiation of any experiments.

Each subject participated in two fMRI scan sessions, during which the following data were acquired: 2 T1-weighted in-plane anatomical scans, 4 baseline scans, 8 functional visual field mapping scans, and 1 T1-weighted anatomical volume.

### Anatomical Data Acquisition and Analysis

Experiments were performed on the 3T Philips Achieva MR scanner at UCI with an 8 channel SENSE imaging head coil. A high-resolution, whole-brain anatomical dataset was acquired for each subject with the following parameters set to maximize the image contrast between white and gray cortical matter: T1-weighted 3D MPRAGE, 1 mm^3^ voxels, TR = 8.4 ms, TE = 3.7 ms, flip = 8°, SENSE factor = 2.4. The anatomical volume was corrected for inhomogeneity and linearly transformed with no rescaling and no distortion to align with the Talairach reference brain, using tools from the FMRIB software library^1^. This high-resolution anatomical dataset was used to identify the gray matter of the cortical sheet for detailed analysis of the functional measurements.

*mrVista* is an open-source, Matlab-based, signal-processing software package developed by the Wandell lab at Stanford University that our lab standardly uses for analysis of individual-subject neuroimaging data^2^. In each subject’s high-resolution anatomical dataset, the location of the cortical white matter was identified – or segmented from the overlying gray matter– using the *mrVista* automated algorithm and expert hand-editing (Teo et al., 1997). The segmented white matter surface was then used to grow a 3–4 mm layer of gray matter. To improve sensitivity and decrease noise, this gray matter was subsequently used to identify which voxels would be analyzed in functional scan measurements (Wandell et al., 2000).

With each functional scan session, one anatomical in-plane dataset was acquired with the same slice prescription as the functional scans but with a higher in-plane spatial resolution (1 mm × 1 mm × 3 mm voxels). As these T1-weighted anatomical images were thus physically in register with the functional scan slices, they were used to align the functional data with the high-resolution anatomical data, first by a manual co-registration and then by a semi-automated, 3D co-registration algorithm, a mutual information method (Maes et al., 1997; Nestares and Heeger, 2000).

### Functional Data Acquisition and Analysis

Functional MR scans were performed on the same scanner as the anatomical data. For each scan, 35 axial slices were acquired that were oriented close to parallel to the superior temporal gyrus for optimal coverage of the cortex (T2-weighted, gradient echo imaging, TR = 2 s, TE = 30 ms, flip = 90°, SENSE factor = 1.7, reconstructed voxel size of 1.875 × 1.875 × 3 mm, no gap). These parameters were used for all visual stimuli functional scans (i.e., moving bars – experimental scans; expanding rings – baseline scans).

Data from each scan were analyzed voxel-by-voxel with no spatial smoothing. To assess data for potential artifacts from head movements, the mean value maps of the blood oxygenation level dependent (BOLD) signal were compared across all scans within one scan session. Motion correction was not needed for any scans in this study, as all scans had less than one voxel of head motion from these experienced subjects. A high-pass filter was applied to the time series from each scan to remove low-frequency sources of physiological noise. The time series from matching scans (i.e., moving-bar stimulus scans) were then averaged together to form one mean time series for that scan type for each subject; this average time series was then used in the pRF modeling analysis. Following registration to the high-resolution anatomy as described above, each subject’s VFM data were displayed on a 3D rendering of that subject’s cortical surface and on a flattened section of the cortical sheet to allow for optimal delineation of VFM boundaries (Wandell et al., 2000).

### Visual Stimulus Presentation: Moving Bars

The Psychophysics Toolbox (Brainard, 1997; Pelli, 1997) in the Matlab programming environment was used to create stimuli on a Dell Optiplex desktop. A Christie DLV1400-DX DLP projector (spatial resolution: 1024 × 768 pixels, refresh rate: 60 Hz) was used to back-project the stimuli onto a display screen at the head end of the bore of the magnet. An angled front-surface mirror was mounted on the scanner head coil close to the eyes to allow the subjects to view the stimuli with a viewing distance of approximately 70 cm. Surgical tape on the subject’s forehead and padding within the head coil were used to minimize head movements.

Visual field maps were measured using a moving-bar stimulus, which was composed of a dynamic-checkerboard, high-contrast pattern with a spatial frequency of 1 cycle/deg and a modulation metameric to the modulation of a ∼500 nm light (luminance = 140 cd/m^2^). The bar aperture spanned a visual field subtending a maximum radius of 11° of visual angle with a width that subtended 1/4th of the stimulus radius. The bar was displaced in discrete steps every 2 s in synchrony with the fMRI volume acquisition. Four bar orientations (0, 45, 90, and 135° from vertical) with two motion directions orthogonal to each orientation were used, producing eight different bar configurations and a total presentation time of 192 s at one cycle/scan (no repetition) (Dumoulin and Wandell, 2008, p. 33). Four mean-luminance periods were inserted in the last 12 s of each 48 s period, at a frequency of 4 cycles/scan. At this rate, each mean-luminance presentation replaces a different position of the stimulus. These mean-luminance periods are used with pRF modeling to allow us to capture visuospatial responses more effectively in regions with large receptive fields, as we expected to measure in pSTS. The mean-luminance stimulus changes the response in a particular voxel in a region with small receptive fields (e.g., V1) only when the mean-luminance replaces the bar stimulus at the preferred visual position for that voxel (Dumoulin and Wandell, 2008). In contrast, responses in regions with large receptive fields [e.g., lateral occipital cortex (LO)] decrease across many/all voxels in the region whenever the mean-luminance period occurs. This baseline measurement provided by the mean-luminance period allows us to distinguish the small pRF sizes in areas like V1 from the large pRF sizes in areas like LO and here in pSTS.

Each column of the checkerboard pattern spanned the length of the bar aperture, and each row spanned its width. Adjacent rows appeared to move in opposite directions to each other with a 2-Hz temporal frequency, and the motion direction changed randomly every 2–3 s. Subjects attended to these moving-bar apertures and responded with a button press – not in sync with the visual stimulus position changes or mean-luminance periods – to an intermittent, subtle change in the motion direction of the checkerboard pattern.

### Population Receptive Field Modeling Analysis: pRF Center and Size Measurements

We used the pRF modeling method to estimate VFM organization and characteristics for each subject, as described here briefly; for complete details of this analysis method, see Dumoulin and Wandell (2008). The population of receptive fields (RFs) in each voxel of retinotopically organized regions of cortex is expected to have similar preferred centers (i.e., location in visual space driving the peak neural responses) and sizes (i.e., the degrees of visual angle driving significant neural responses), allowing their combined population RFs (pRFs) to be estimated as a single, two-dimensional (2D) Gaussian RF. For each voxel independently in an individual subject’s dataset, the BOLD response to the moving-bar stimulus was predicted by iteratively testing a wide range of possible 2D Gaussian pRFs with parameters of preferred center location (x, y), which are used to determine the traditional eccentricity and polar-angle responses, and spread (σ; standard deviation of the 2D Gaussian), which is used to describe pRF size). The predicted fMRI time series was calculated by convolving the stimulus sequence and BOLD hemodynamic response function (HRF) with each tested pRF (Boynton et al., 1996; Friston et al., 1998). The pRF parameters of position and size ultimately assigned to each voxel minimized the sum of squared errors between the observed and predicted fMRI time series.

As a primary measurement of goodness of fit, each voxel was independently assessed in terms of its percent variance explained, which we converted here to coherence values for comparison with the phase-encoded traveling wave methods used for our baseline measurements (Barton and Brewer, 2015) as well as numerous previous visual field mapping studies (for reviews, see Wandell et al., 2007; Brewer and Barton, 2012b). All voxels considered for further analysis displayed statistically significant responses to the moving-bar stimulus, with a coherence measurement of the BOLD signal of at least 0.25 (see section below for further discussion of coherence measurements). Eccentricity 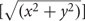 and polar angle [tan^-1^(y/x)] coordinates were derived from the 2D Gaussian pRF models and were used to plot the VFM representations on the cortical surface for each subject. The pRF sizes were measured as a function of eccentricity for each VFM, collapsed across subjects. As with all visual field mapping measurements, there may be interactions in the most peripheral measurements with large receptive fields centered in the further periphery that overlap the stimulus edge. These interactions may affect the pRF center and size measurements in complicated ways in the most peripheral measurements; thus, we routinely crop our data in by at least 1°. In this study, we only report measurements for eccentricity bins including data out to 10° of eccentricity rather than the full 11° radius of the stimulus.

### Coherence Measurements

A traditional measure of statistical significance used in visual field mapping, coherence is equal to the amplitude of the BOLD signal modulation at the stimulus frequency divided by the square root of the power of the BOLD modulation at all other frequencies except the first and second harmonics (Engel et al., 1994, 1997; Wandell et al., 2007; Brewer and Barton, 2012b, 2014). Here, coherence was converted from the variance explained of the pRF model fit (Barton and Brewer, 2015). Our coherence threshold of 0.25 is on the conservative side of statistical significance thresholds in visual field mapping studies. For this study, we also empirically validated this threshold by measuring the coherence of the noise activity elicited by our baseline stimulus (see below for details) (Barton and Brewer, 2015).

### Definition of Visual Field Maps

We define a VFM in accordance with very exact criteria: (i) a VFM comprises two orthogonal, non-repeating topographical representations of visual space: eccentricity and polar angle; (ii) each of these topographical representations must be organized as a generally contiguous, orderly gradient; (iii) each VFM should represent a substantial portion of sensory space – e.g., a hemifield of the visual field (VFMs vary in the degree to which their pRFs extend into ipsilateral space, so we ignore extent of ipsilateral representation in this definition); and (iv) the general features of each VFM should be consistent across individuals (Sereno et al., 1995a; DeYoe et al., 1996; Van Essen, 2003; Brewer et al., 2005; Wandell et al., 2007; Brewer and Barton, 2012b).

### Probability of Spurious Gradients Emerging from Noise

An eccentricity or polar-angle gradient is one of the most highly organized features of the cortical surface ever measured using fMRI. Such gradients are identified by trained experts using colors that represent stimulus values because human visual pattern recognition is still necessary, even with the best efforts to date to perform computer-based atlas fitting (Dougherty et al., 2003; Brewer and Barton, 2012b). The fact that human recognition is involved has led to a critique that researchers are “reading tea leaves” when performing gradient identification, implying that it would be too easy for a biased researcher to mistake spurious gradients arising from noise for signal from a true gradient. Thus, it is important to estimate just how unlikely it is for an organized gradient to arise from noise in an area of cortical surface the size of a VFM. In other words, we can estimate the probability that a VFM gradient is observed under the null hypothesis that pRF centers are distributed randomly.

**FIGURE 1:**
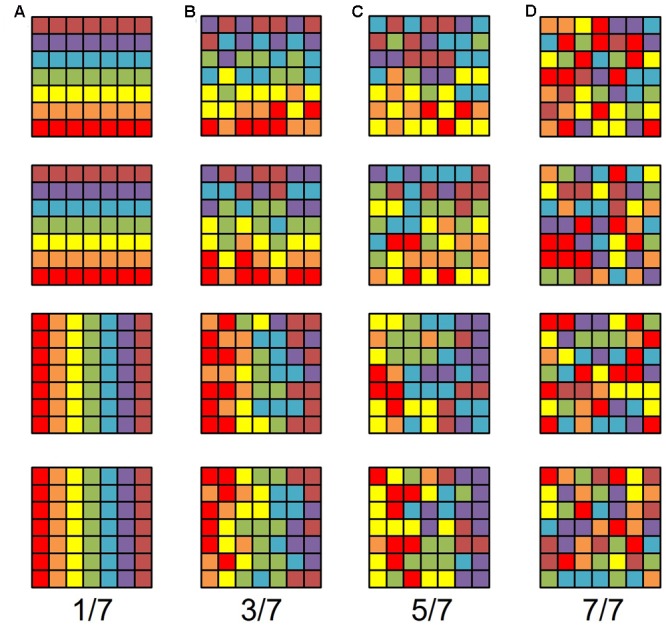
**Model gradients.** Each row represents one sensory gradient evenly distributed across a piece of cortical surface consisting of a 7 × 7 matrix of voxels. The color code is such that each color represents a stimulus value within 1/7 of the overall stimulus space, ranging from the lowest value in red, in order to the highest value in brown. For example, red would represent an eccentricity preference from fixation value 0.00–1.57° of visual angle with our 11°-radius bar stimulus, green would be 4.71–6.28°, and brown would be 9.43–11.00°. Each column represents gradients with a different amount of random noise in each voxel, such that there is no random noise in the left-most column and completely random noise in the right-most column. In other words, the acceptable noise for each voxel is zero in the leftmost column and looser as one moves rightward. In **(A)**, each voxel represents exactly 1/7th of the stimulus space that it should (e.g., if the voxel should be green, it is, without variation). In **(B)**, if a voxel should represent a particular 1/7th of the stimulus range in the gradient, it can with equal probability represent an adjacent color, such that the true value falls somewhere within 3/7th of the stimulus range, centered on the correct value (e.g., if a voxel should be green, it can be yellow, green, or blue with equal probability). In **(C)**, if a voxel should represent a particular 1/7th of the stimulus range in the gradient, it can with equal probability represent 5/7th of the stimulus range, centered on the correct value (e.g., if a voxel should be green, it can be tan, yellow, green, blue, or purple, with equal probability). In **(D)**, it doesn’t matter what 1/7th of the stimulus space the voxel should represent given the gradient, any color can be assigned to each voxel, with equal probability. Note that gradients in **(B)** are still identifiable as the same gradients in **(A)**, despite significant noise. Note also that the gradients in **(C)** have more structure than completely random noise in **(D)**, but are visibly less orderly than the gradients in **(B)**.

For such a gradient to arise, a large number of voxels must be organized such that they span an entire range of pRF centers in sensory space, in order from one boundary to the other (from lowest to highest eccentricity or from upper to lower vertical meridian for polar angle). An eccentricity gradient can be modeled as an M by N matrix of voxels, where each column spans the entire range of eccentricities (**Figure 1**). For simplicity, we consider the case where the eccentricity representation is evenly distributed across the M rows of each column, such that each row in a gradient represents 1/M of the total eccentricity range *E_min_* to *E_max_*. Let each voxel’s eccentricity value e be drawn from distribution E ∼ uniform (*E_min_, E_max_*). Eccentricity values for voxels in rows R_1_, …, R_M_ must fall within intervals [*E_min_* + (R – 1)^∗^ ((*E_max_ – E_min_*) / M), E_min_ + R ^∗^ ((*E_max_ – E_min_*)/M)]. The probability of observing a voxel with eccentricity value e appropriate for its row is therefore 1/M. The probability p of observing all voxels which meet the criteria for an eccentricity gradient is therefore (1/M)^(M^∗^N)^. It follows similarly for a polar angle (or other sensory) gradient.

For a conservative estimate, we chose a VFM surface area of ∼220 mm^2^, which is approximately the smallest reported size of individual-subject measurements for the V3v quarterfield (Dougherty et al., 2003; Brewer and Barton, 2014). Each voxel measures 1.875 mm × 1.875 mm × 3 mm. Assuming an equal number of voxels contribute their 1.875 mm × 1.875 mm (3.52 mm^2^) and 1.875 mm × 3 mm (5.625 mm^2^) sides to the VFM surface area, the effective surface area per voxel is 4.57 mm^2^. We can approximate the mean VFM with a 7 × 7 matrix of 4.57 mm^2^ units with a total surface area of 224 mm^2^. Therefore, the probability of observing either an eccentricity or polar-angle gradient in that cortical area is (1/7)^(7∗7)^, or 3.89^∗^ 10^-42^ (**Figure 1A**).

Of course, that scenario entails little tolerance for error. In reality, no gradient is perfect, and it is actually the human experts’ ability to see gradients through some noise that gives them the advantage over computer-based atlas fitting. Consider the case where each voxels’ acceptable range of eccentricity values is tripled from 1/M to 3/M. The probability p of a gradient arising from noise keeping the other parameters the same becomes (3/7)^(7^∗^7)^, or 9.31 ^∗^ 10^-19^, which is much higher than the 1/M case, but still remote. Note that an example 3/M gradient is rough, but still identifiable (**Figure 1B**). We think that this should form a rough upper limit on the acceptable error. As such, that probability is what we use for observing a gradient given the null hypothesis that the gradient emerged spontaneously from random noise. Even a higher amount of “allowed error,” quintupling from 1/M to 5/M, yields a remote probability of a spurious gradient (5/7)^(7^∗^7)^, or 6.91 ^∗^ 10^-8^. Note that there is little resemblance between **Figures 1A,C**; at best, the example 5/M gradient is a noisy bifurcation of the stimulus space, and therefore too liberal an estimate of acceptable error.

At this point, five further details about the probability estimate require discussion. First, all the voxels considered above are significantly active voxels, which means that all estimated probabilities just discussed are given that some sort of pRF model fit was possible for the voxels in the first place. In other words, these voxels are systematically, reliably activated by standard visual field mapping stimuli, and it is only the interpretation of the pattern of these significantly active voxels that is addressed by this probability estimate.

Second, the probability estimate assumes that each voxel’s pRF model fit is independent of its neighbors. This assumption could be violated by the model itself, but because each voxel is evaluated independently, with no smoothing of any kind, and no motion correction, the pRF model fit of one voxel has no influence on its neighbors (Dumoulin and Wandell, 2008; Brewer and Barton, 2012b). This assumption could also be violated by the fact that we are measuring BOLD activity in each voxel. In visual cortex, the point spread function is estimated to be 1–2 voxels (3.5 mm), indicating that the raw activity of one voxel leaks somewhat to its neighbors (Engel et al., 1997). However, under the null hypothesis of our probability estimate, a group of voxels with random stimulus preferences would influence their neighbors randomly as well, which would not meaningfully change our probability estimates.

Third, we performed the calculation assuming an even distribution of pRF fits across the stimulus space, but VFMs along dorsal and ventral pathways, for example, have been shown to have clear differences in cortical magnification (e.g., Brainard, 1997; Kastner et al., 2001; Press et al., 2001; Huk et al., 2002; Dougherty et al., 2003; Brewer et al., 2005; Wandell et al., 2007; Brewer and Barton, 2012b). In such cases, the number of rows is larger than the number of evenly divided intervals, meaning that the exponential component M is now larger than the denominator of 1/M or 3/M, above. Thus, our probability estimate of a gradient arising from random noise is likely too high, and therefore more conservative.

Fourth, our estimation is based on a rectangle, and VFMs typically are trapezoidal or shaped like a pie slice. Our estimates are meant to be just that, but even if the “columns” of our model do not form a perfect rectangle, but broaden toward the periphery, the difference in probability is marginal, because most surface area lost on one end is gained on the other.

Fifth, so far we have only discussed one gradient arising in a cortical area, but two are necessary to define a VFM hemifield. The odds of two gradients arising in one hemisphere using the 3/M model for this size of VFM, are (9.31 ^∗^ 10^-19^)^2^, or 8.67 ^∗^ 10^-37^. Thus, the probability of measuring 2 gradients using the 3/M model for a VFM with a surface area of approximately 224 mm^2^ in each of 10 hemispheres, as we report for each of the VFMs presently measured, is (8.67 ^∗^ 10^-37^)^10^, or 8.67 ^∗^ 10^-370^.

### Determination of Orthogonality

As noted above, each VFM is expected to represent the contralateral hemifield and be organized such that it contains two orthogonal gradients of visual space representation: one of eccentricity, one of polar angle. The eccentricity and polar-angle gradients must be orthogonal to one another in order to create a complete representation of a contralateral hemifield of visual space. Two such hemifields, one in each hemisphere, then form a complete VFM. To measure whether two such gradients are orthogonal to one another, we identified a vector from the center of mass of the lowest eccentricity from fixation to the highest (an eccentricity vector) along a constant, iso-angle line and from the lower vertical meridian to the upper vertical meridian (a polar-angle vector) along a constant, iso-eccentricity line on the flattened cortical surface and computed the rotational offset between the two vectors (Barton et al., 2012). VFMs with orthogonal gradients (i) should have vectors offset rotationally by about 90° from one another, whereas randomly distributed gradients (ii) should have no consistent arrangement (high standard error, no meaningful average), parallel gradients should have no difference (0°) in vector offset (iii) and anti-parallel gradients should be 180° offset (iv).

### Visual Field Coverage and Population Receptive Field Concentration

The coverage of visual space in a particular VFM is what portion of visual space evokes significant responses from the voxels composing that VFM. The concentration of pRFs for a given VFM is then defined as the number of pRFs within that map that respond to each portion of visual space (Dumoulin and Wandell, 2008; Amano et al., 2009). To identify these two aspects of each VFM, we first identify the pRF coverage of each voxel within a VFM for each visual location. Across the visual field, we assign a binary value of 1 or 0 for each voxel in that VFM, where 1 denotes the visual position as being within the pRF for that voxel, and 0 denotes that location as being outside of that voxel’s pRF. These values are summed and normalized within VFMs to a value between 0 and 1, where 1 corresponds to the highest concentration of pRFs representing that visual field position and 0 to no pRF coverage at all. An image plotting visual space is then created using these normalized values for each spatial location, coded by a colormap. Values above 0 (cyan to red) indicate at least some pRF coverage at a given spatial location, with increasing values indicating more pRFs covering that location, to a maximum concentration for that particular map, at 1 (bright red). Visual field coverage plots thus provide an improved visualization of which portions of visual space are represented by a particular VFM with information about both the pRF center and the spread (coverage across visual space by the group of neurons within each voxel).

### Surface Area Measurements

To measure surface area, the boundaries of each VFM were first identified on a 2D flattened region representing the cortical surface of an individual hemisphere, as described above. Because the process of flattening a section of the cortical sheet unavoidably distorts distance and area measurements, the 2D coordinates were first mapped back to the 3D manifold. The surface area was then measured along the 3D cortical manifold at the division between gray and white matter, which allows more reliable boundary definition than the outer surface of the gray matter or any particular cortical layer (Teo et al., 1997; Wandell et al., 2000). For extended details about the algorithm used in these measurements, see (Dougherty et al., 2003). The surface area of each VFM was measured in individual subjects and then averaged across subjects by VFM.

We also characterized each VFM across subjects in terms of the average percentage of the surface area as a function of eccentricity from fixation. The measurement of the distribution of surface area using pRF centers representing particular regions of visual space is analogous to the measurement of cortical magnification, but takes into account not only the dimension of magnification along iso-angle lines, as does cortical magnification, but also along iso-eccentricity lines (Dougherty et al., 2003; Brewer and Barton, 2012b, 2014).

### Baseline Stimulus: Expanding Rings under Scotopic Conditions

In order to empirically validate the coherence threshold used in the VFM measurements, we also measured cortical responses to a baseline stimulus composed of expanding rings presented under low light – scotopic – conditions (Barton and Brewer, 2015). Scotopic conditions during scanning were created by blacking out all light sources in the scanner room and placing neutral density filters over the projector’s wave guide to achieve a luminance of 0.003 cd/m^2^. Before the baseline scans, subjects dark-adapted for 35–40 min, and all subjects reported being unable to perceive the stimuli for rings within the central 3° of visual angle. Under scotopic conditions, the cortical regions representing the central 3° of visual angle receive little-to-no stimulation, as this luminance level is too low to activate the cone-only fovea (Hadjikhani and Tootell, 2000; Baseler et al., 2002; Dougherty et al., 2003; Duffy and Hubel, 2007; Hubel et al., 2009).

The expanding-ring stimulus comprised the same high-contrast, flickering, black and white checkerboard patterns as the moving-bar stimulus, but subtended only a maximum radius of 3° of visual angle. The checkerboard was organized as a dartboard pattern with a radial spatial frequency of 5 cycles/deg and an angular frequency of 12 cycles/2π. Each stimulus had a 45° duty cycle, spanning appropriate eccentricities in 12 steps. Seven cycles were completed in each scan, creating a 7 cycles/scan traveling wave of activity. No mean-luminance periods were inserted. Other details of the ring stimulus matched that of the moving-bar stimulus.

Subjects maintained fixation on a large central cross that spanned the diagonals from each corner of the field of view. The lines of the fixation cross were approximately 0.5° thick and were present throughout each scan. This thin, large fixation cross allows subjects to maintain fixation under scotopic conditions under which the central 3° of visual angle – the fovea – is not activated. Subjects were instructed to perform the same motion perception attentional task as for the moving-bar stimulus. As the subjects could not perceive the stimulus, they could not successfully perform this task, but it served to engage the subjects’ attention to the central 3° of their visual fields. Subjects were trained on this type of fixation and this attention task with identical stimuli under photopic conditions (luminance = 140 cd/ m^2^) and with other scotopic scans in which the visual stimuli spanned 7.4° and 11° of visual angle and thus were visible outside of the foveal region (Barton and Brewer, 2015).

### Baseline Data Analysis

Field-standard, visual field mapping, traveling-wave analyses were performed on the baseline stimuli using *mrVista* and the same functional data analysis as described above (for additional discussion, see Engel et al., 1994; Sereno et al., 1994, 1995a; DeYoe et al., 1996; Brewer et al., 2002; Dougherty et al., 2003; Brewer et al., 2005; Wandell et al., 2007; Brewer and Barton, 2012b). Our measurements show a maximum baseline noise level for coherence from these traveling wave measurements of 0.15, well below our statistical threshold of coherence (co) = 0.25 used for the moving-bar pRF analysis.

## Results

### V3A/B Cluster

**FIGURE 2:**
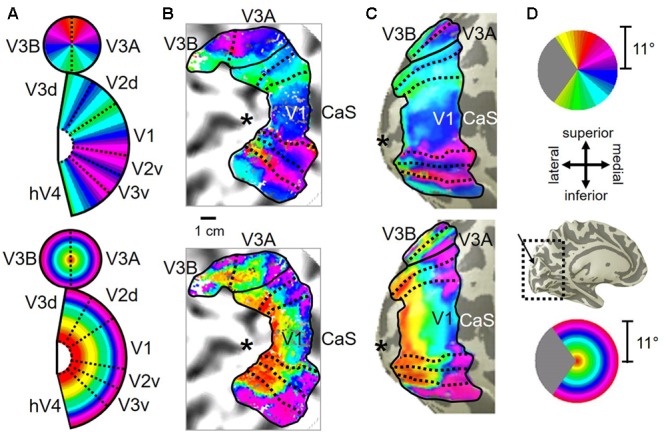
**V3A/B cluster.** The colors in the schematic **(A)** and the pseudocolor overlays on the flattened **(B)** and inflated **(C)** representations of cortex represent the position in visual space that produces the strongest response at that cortical location [see color legends in **(D)**]. Solid black lines indicate VFM boundaries between VFMs along peripheral eccentricity reversals, which separate cloverleaf clusters from one another. Dotted black lines indicate VFM boundaries between maps along polar angle reversals, which separate maps within cloverleaf clusters. Scale bar denotes 1 cm along the flattened cortical surface. **(A)** *Top:* Schematic model of the polar angle aspect of the VFMs; *Bottom:* Schematic model of the eccentricity aspect of the VFMs. **(B)** *Top:* The polar angle aspect of the VFMs on a flattened representation of cortex in a single left hemisphere; *Bottom:* The eccentricity aspect of the VFMs on the same flattened representation of cortex. **(C)** *Top:* The polar angle aspect of the VFMs on an inflated mesh representation of cortex in a single left hemisphere; *Bottom:* The eccentricity aspect of the VFMs on the same inflated view. **(D)** *Top:* Color legend for polar angle representations; *Middle/Top:* Orientation legend; *Middle/Bottom:* Inflated mesh brain, indicating the cropped view shown in **(C)**. Arrow points to approximate location of V3A and V3B; *Bottom:* Color legend for eccentricity representations. Coherence ≥ 0.25. All data for **(A–D)** are from the left hemisphere of S2. **(B,C)** Asterisk denotes the approximate location of the occipital pole.

**FIGURE 3:**
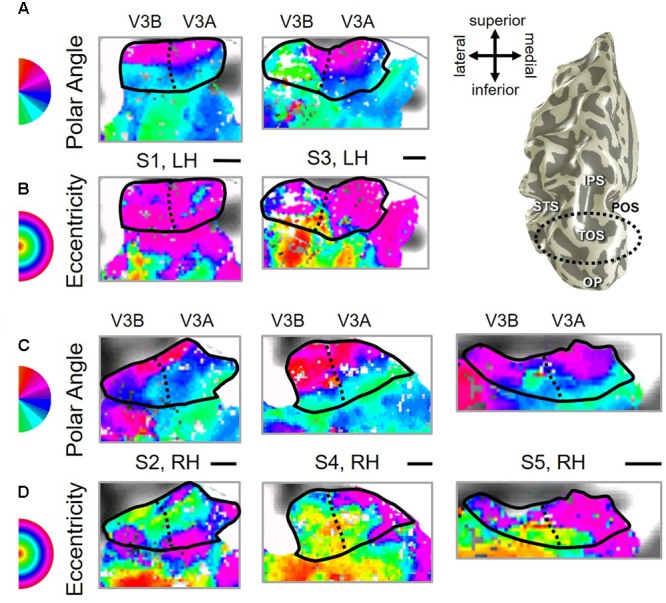
**Examples of the V3A/B cluster across subjects. (A,B)** The V3A/B cluster in left hemispheres (LH) from S1 and S3. **(C,D)** The V3A/B cluster in right hemispheres (RH) from S2, S4, and S5. **(A,C)** V3A/B cluster polar angle representations on flattened representations of cortex. **(B,D)** V3A/B cluster eccentricity representations on the same flattened representations of cortex as pictured directly above in **(A,C)**. Scale bars are shown for each subject’s flattened cortex and denote 1 cm. Inset in top right displays approximate anatomical directions for the flattened cortices and the cluster’s location on a representative 3D left hemisphere. Note that left and right hemispheres have been aligned to match in orientation. The V3A/B cluster typically lies along the transverse occipital sulcus (TOS; dotted black line) at the base of the intraparietal sulcus (IPS). STS: superior temporal sulcus. POS: parietal occipital sulcus. Other details are as in **Figure 2**.

**FIGURE 4:**
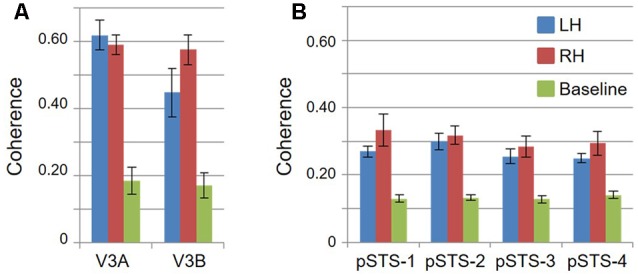
**Coherence measurements.** Graphs display the coherence measurements in each VFM for visual bars and baseline stimuli, indicating signal and noise activity, respectively. All data are averaged across hemispheres (i.e., 5 hemispheres for left and right visual bar data; 10 hemispheres for baseline data). **(A)** VFMs in V3A/B cluster. **(B)** VFMs in pSTS cluster. Blue: left hemisphere (LH); Red: right hemisphere (RH); Green: baseline. Error bars are SEM.

Consistent with previous work, our VFM measurements demonstrated the presence of the V3A/B cloverleaf cluster located dorsally, adjacent to V3d and/or LO-1 along the TOS (**Figures 2, 3** and see **Table 1** for Talairach coordinates) (Smith et al., 1998; Press et al., 2001; Brewer et al., 2005; Tyler and Wade, 2005; Wandell et al., 2005; Larsson and Heeger, 2006; Brewer and Barton, 2012b). The average coherence of our measurements of each of these two VFMs was far above our chosen coherence threshold (co ≥ 0.25) and measured baseline noise (co ≤ 0.15) (**Figure 4**). V3A and V3B form a radially orthogonal cluster with the foveal representation in the center and increasingly peripheral representations along the outer sections of the cluster. Because the V3A/B cluster contains only two VFMs, the visual field representations appear to curve to split a ventral peripheral eccentricity reversal, a lower vertical meridian polar angle reversal, or both with V3d and/or LO-1 (Brewer et al., 2005; Tyler and Wade, 2005; Wandell et al., 2005). V3A is positioned along the medial aspect of the TOS and shares the dorsal border of V3d (**Figure 2**). V3B joins V3A at the foveal representation and extends peripherally across the lateral aspect of the TOS. The exact position of V3B in relation to the dorsal border of V3d varies across subjects; V3B shares a border as described above with only V3d, with V3d and LO-1, or with only LO-1, depending on the size and location of the other VFMs in the visual hierarchy of each subject (Press et al., 2001; Larsson and Heeger, 2006). The dorsal portions of each VFM represent the upper visual quarterfield and border the posterior parietal VFMs along the IPS (Silver et al., 2005; Swisher et al., 2007; Silver and Kastner, 2009).

**Table 1:** Talairach coordinates.

	Left hemisphere			Right hemisphere		
Map	*x*	*y*	*z*	*x*	*y*	*z*
V3A/B	–27.2 ± 1.8	–89.2 ± 1.7	15.8 ± 2.7	20.2 ± 3.9	–89.0 ± 1.6	20.6 ± 4.6
V3A	–21.0 ± 1.3	–89.8 ± 2.1	14.6 ± 2.6	16.8 ± 4.2	–90.8 ± 1.8	23.0 ± 4.4
V3B	–31.0 ± 2.2	–88.2 ± 1.7	16.0 ± 3.0	25.2 ± 2.7	–86.4 ± 2.0	16.8 ± 4.2
pSTS	–55.5 ± 3.8	–46.8 ± 3.8	7.5 ± 1.2	51.8 ± 3.0	–48.6 ± 4.6	15.2 ± 3.5
pSTS-1	–50.3 ± 3.5	–49.3 ± 2.5	8.8 ± 0.5	48.0 ± 2.5	–49.4 ± 5.8	16.0 ± 3.6
pSTS-2	–55.3 ± 3.4	–44.5 ± 4.3	7.8 ± 0.5	50.8 ± 2.6	–47.4 ± 3.9	16.8 ± 3.9
pSTS-3	–56.8 ± 3.3	–45.8 ± 5.8	6.0 ± 2.7	53.0 ± 3.5	–46.6 ± 3.8	14.4 ± 3.8
pSTS-4	–53.8 ± 4.4	–49.3 ± 3.1	8.5 ± 1.9	50.6 ± 3.2	–49.6 ± 5.9	14.2 ± 3.7

*The Talairach coordinates were averaged across the left and right hemispheres (*N* = 5 subjects) and presented for each cloverleaf cluster and VFM.*

**FIGURE 5:**
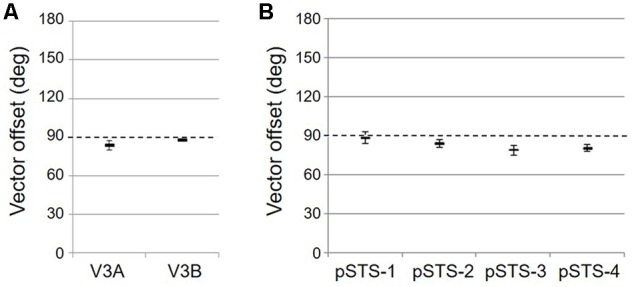
**Results of the vector-offset orthogonality test for each VFM.** Offsets near 90° represent orthogonal eccentricity and polar-angle gradients. **(A)** VFMs in the V3A/B cluster. **(B)** VFMs in the pSTS cluster. All data are averaged across all 10 hemispheres. Error bars are SEM.

**FIGURE 6:**
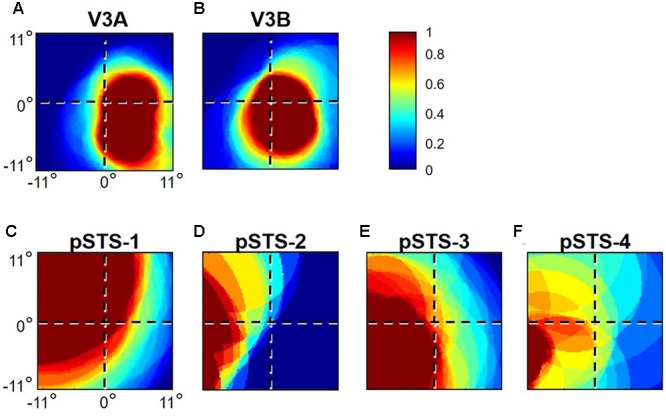
**pRF coverage of visual space in the V3A/B and pSTS clusters.** Concentrations of pRFs across the central 11° radius of visual space for **(A)** V3A and **(B)** V3B are shown for S2’s left hemisphere. Corresponding V3A/B-cluster VFMs are displayed in **Figure 2**. Inset (*top right*): color legend indicates the normalized pRF concentration from high in red to no coverage at all in dark blue. Concentrations of pRFs across the central 11° radius of visual space are also shown for S1’s right pSTS cluster: **(C)** pSTS-1; **(D)** pSTS-2; **(E)** pSTS-3; **(F)** pSTS-4. Corresponding pSTS-cluster VFMs are displayed in **Figure 10**.

As illustrated in **Figure 5A**, the low-to-high vectors for each gradient were reliably offset by roughly 90° in both VFMs, confirming that each VFM contains orthogonal eccentricity and polar-angle gradients. We were able to clearly identify two orthogonal gradients to define both V3A and V3B in all 10 hemispheres of our five subjects, and all VFMs contained hemifield representations of the contralateral visual field (**Table 2**). As seen in the visual field coverage plots of **Figures 6A,B**, the centers and spreads of all the significantly active voxels (co ≥ 0.25) fell within the contralateral visual field with a little overlap into the ipsilateral field along the vertical meridian especially near the fovea, as expected for these regions (Baizer et al., 1991; Ejima et al., 2003).

**Table 2:** Visual space coverage.

Map	Across all hemispheres			
	Complete	Lower	Upper	Total
V3A/B	10	0	0	20/20
V3A	10	0	0	10/10
V3B	10	0	0	10/10
pSTS	26	6	4	36/40
pSTS-1	7	1	1	9/10
pSTS-2	7	1	1	9/10
pSTS-3	6	2	1	9/10
pSTS-4	6	2	1	9/10

*For visual space coverage, “complete” indicates the number of times the VFM (or maps within a cluster) represented a complete contralateral hemifield in roughly equal proportions between the upper and lower quarterfield. “Lower” indicates the number of times the VFM (or maps within a cluster) represented primarily the lower contralateral quarterfield. “Upper” indicates the number of times the VFM (or maps within a cluster) represented primarily the upper contralateral quarterfield. “Total” indicates the number of times the VFM (or maps within a cluster) represented at least one contralateral field. For visual space coverage, data was summed across all 10 hemispheres.*

**FIGURE 7:**
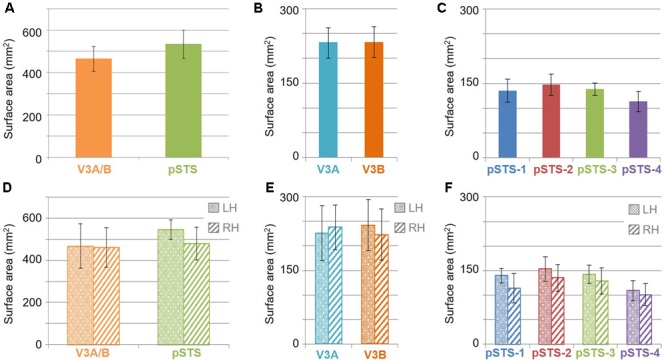
**Surface area measurements. (A)** Total surface area for each cloverleaf cluster. Orange: V3A/B; Green: pSTS. **(B)** Total surface area for V3A (*dark orange*) and V3B (*teal*) VFMs. **(C)** Total surface area for each pSTS VFM. Blue: pSTS-1; Red: pSTS-2; Green: pSTS-3; Purple: pSTS-4. **(D)** Total surface area for each cloverleaf cluster by hemisphere. **(E)** Total surface area for V3A and V3B by hemisphere. **(F)** Total surface area for each pSTS VFM by hemisphere. Note the change in y-axes between **(A,D)** and **(B,C,E,F)**, altered for improved legibility. LH: left hemisphere, dotted bars; RH: right hemisphere, striped bars. All data are averaged across all subjects. Error bars are SEM.

The surface area of the full V3A/B cluster ranged across subjects from 308 mm^2^ to 628 mm^2^ (mean: 464 mm^2^; SEM: 59 mm^2^; **Figure 7A** and **Table 3**). This average cluster surface area is similar to that reported for the V3d quarterfield (Dougherty et al., 2003). The V3A and V3B maps were very similar in size to each other within subjects, and the average sizes across subjects and hemispheres were nearly the same (**Figures 7B,D**), with an average area of 232 mm^2^ for V3A (SEM: 29 mm^2^) and 233 mm^2^ for V3B (SEM: 31 mm^2^). There were no significant differences in surface area between hemispheres for each VFM (**Figure 7E**; V3A: *p* = 0.89; V3B: *p* = 0.83). Given these mean surface areas for V3A and V3B, our example probability calculation for a VFM with a surface area of 220 mm^2^ can be used for these maps. We thus reject the null hypothesis that the two gradients in each V3A/B VFM observed in 10 hemispheres arose out of random noise.

**Table 3:** Surface area measurements.

	Surface area (mm^2^)						
Map	S1	S2	S3	S4	S5	Avg.	SEM
V3A/B	308	628	375.5	569	441.5	464	59
LH	310	832	284	325	588	468	106
RH	306	424	467	813	295	461	94
V3A	137.5	306.5	210	280.5	225	232	29
LH	114	404	129	173	309	226	56
RH	161	209	291	388	141	238	46
V3B	170.5	321.5	165.5	288.5	216.5	233	31
LH	196	428	155	152	279	242	52
RH	145	215	176	425	154	223	52
pSTS	497.5	585.5	436	388	764	534	66
LH	553	667	513	447	—	545	46
RH	442	504	359	329	764	480	77
pSTS-1	112	146.5	85.5	110.5	222	135	24
LH	161	168	112	118	—	140	14
RH	63	125	59	103	222	114	30
pSTS-2	116.5	182.5	177	78.5	182	147	21
LH	159	162	207	86	—	154	25
RH	74	203	147	71	182	135	27
pSTS-3	157	142	105.5	116.5	171	138	12
LH	102	192	142	133	—	142	19
RH	212	92	69	100	171	129	27
pSTS-4	112	114.5	68	82.5	189	113	21
LH	131	145	52	110	—	110	20
RH	93	84	84	55	189	101	23

*The table presents measurements of total surface area in mm^2^ for clusters and VFMs as well as surface area by hemisphere for each. Individual-subject measurements are shown for columns S1–S5. Right two columns display average surface area and SEM. ‘—’ denotes that there were no topographic responses above the coherence threshold for that VFM/cluster (S5, LH). LH, left hemisphere; RH, right hemisphere.*

**FIGURE 8:**
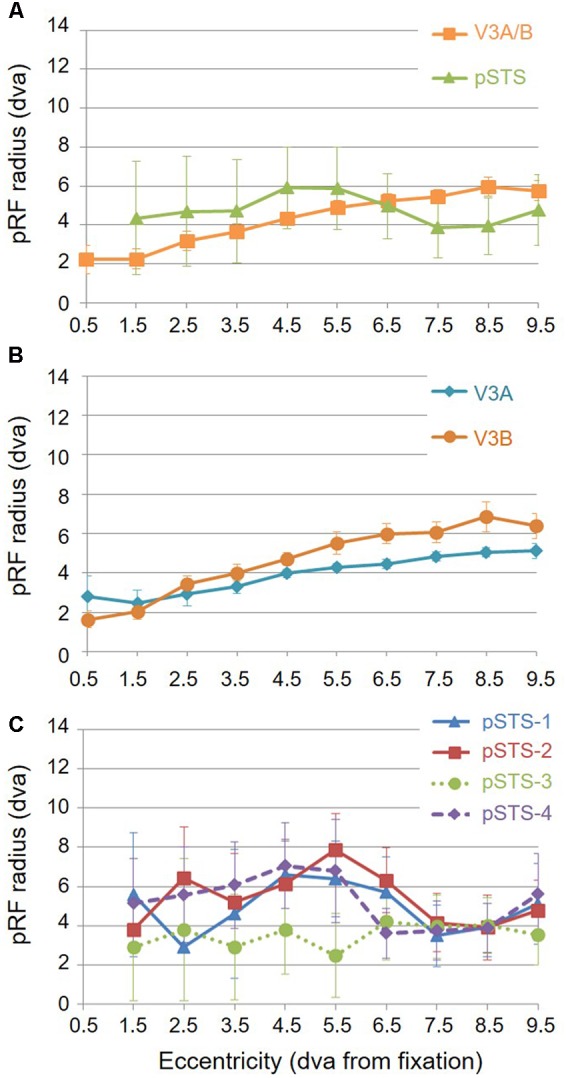
**Average pRF sizes.** Graphs depict the average pRF radius as a function of 1° eccentricity bins from fixation to 10° for each VFM. Note that there are no pRF centers that fall within the 0.5 eccentricity bin due to the relatively large pRF sizes within the pSTS cluster (Brewer and Barton, 2014, p. 212). **(A)** pRF sizes for each cloverleaf cluster. Orange squares: V3A/B; Green triangles: pSTS. **(B)** pRF sizes for V3A and V3B VFMs. Dark orange circles: V3A; Teal diamonds: V3B. **(C)** pRF sizes for each pSTS VFM. Blue triangles: pSTS-1; Red squares: pSTS-2; Green circles, dotted line: pSTS-3; Purple diamonds, dashed line: pSTS-4. All data are averaged across all 10 hemispheres. dva, degrees of visual angle. Error bars are SEM.

**FIGURE 9:**
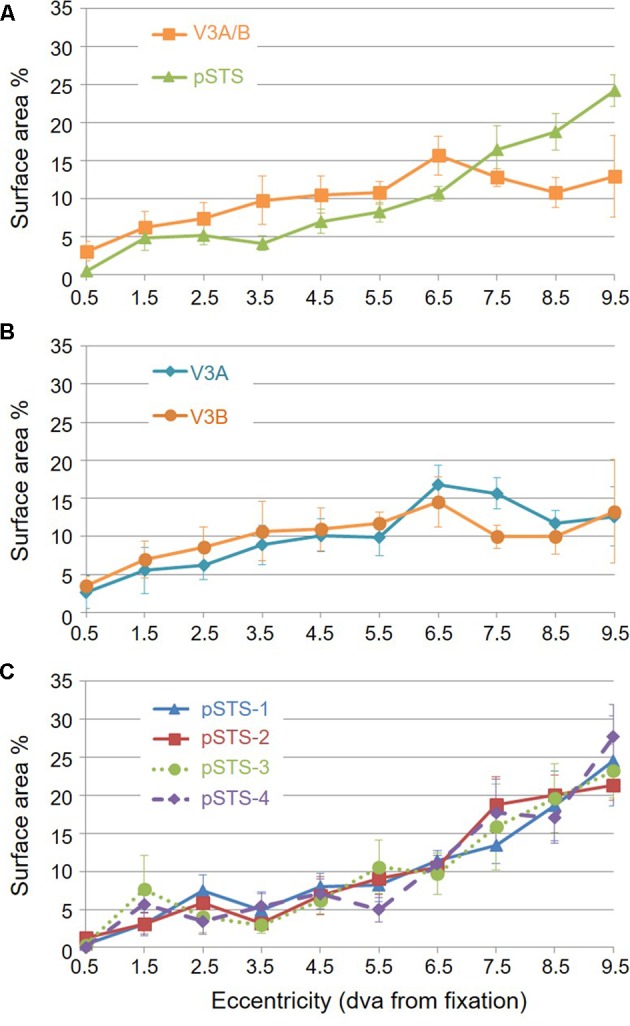
**pRF center distributions.** Graphs depict distributions of pRF centers’ surface area by 1° eccentricity bins from fixation to 10° for each VFM, as a percent of the total surface area devoted to visual space within the central 10° of visual angle. Note that there are no pRF centers that fall within the 0.5 eccentricity bin – and thus 0% surface area distributed here – due to the relatively large pRF sizes within the pSTS cluster (Brewer and Barton, 2014, p. 212). **(A)** Distribution for each cloverleaf cluster. Orange squares: V3A/B; Green triangles: pSTS. **(B)** Distribution for V3A and V3B VFMs. Dark orange circles: V3A; Teal diamonds: V3B. **(C)** Distribution for each pSTS VFM. Blue triangles: pSTS-1; Red squares: pSTS-2; Green circles, dotted line: pSTS-3; Purple diamonds, dashed line: pSTS-4. All data are averaged across all 10 hemispheres. dva, degrees of visual angle. Error bars are SEM.

For analysis of the measurements of pRF sizes and the distribution of the representation of visual space (i.e., % surface area distribution), we divided up the eccentricity representation in each VFM in each hemisphere of each subject into 10 regions of interest (ROIs) spanning 1° of visual angle along the eccentricity gradient from 0 to 10°, centered on every half degree. Each measurement was drawn from these 10 eccentricity-band ROIs for each subject and then averaged across hemispheres and subjects. We found that V3A and V3B exhibited increasing pRF sizes as a function of eccentricity (**Figures 8A,B**); this is similar to the V1-hV4 VFMs in early and mid-level visual cortex, which have generally small pRF sizes that increase more eccentric from fixation (Smith et al., 2001; Dumoulin and Wandell, 2008; Barton and Brewer, 2015). The average pRF radius for the cluster ranged from ∼2° in the measurements of the more foveal eccentricities to ∼6° in the measurements of the more peripheral eccentricities (**Figure 8A**). V3B had slightly larger pRF sizes (∼1–2°) than V3A for the peripheral half of the measurements (5–10° of eccentricity). Conversely, the percentage of the surface area increases more eccentric from fixation, demonstrating a larger representation of the more peripheral regions (5–10°) that is remarkably different from the foveal expansion of the V1-hV4 VMFs (**Figure 9**) (Dougherty et al., 2003; Ejima et al., 2003; Brewer et al., 2005; Wandell et al., 2007; Brewer and Barton, 2012a). These measurements are consistent with the expectations for expanded peripheral representations of visual space along the mid- and high-level regions of the dorsal stream (Baizer et al., 1991; Huk et al., 2002; Swisher et al., 2007; Kolster et al., 2009, 2010; Silver and Kastner, 2009).

### Posterior Superior Temporal Sulcus Cluster

**FIGURE 10:**
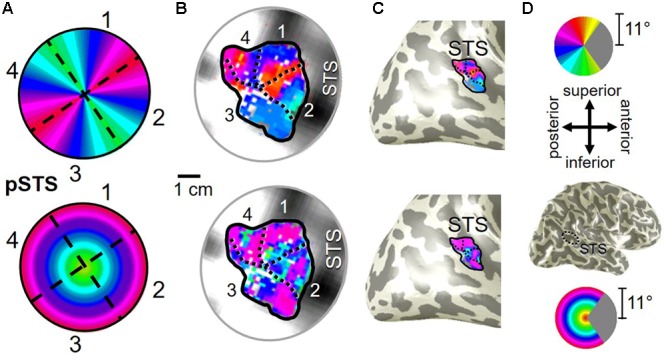
**Posterior superior temporal sulcus (pSTS) cluster.** All data for **(A–D)** are from the right hemisphere of Subject 1. Numbers in **(A)** refer to visual field map numbers within the pSTS cluster (pSTS-1, pSTS-2, pSTS-3, and pSTS-4). STS: superior temporal sulcus. Other details as in **Figure 2**.

**FIGURE 11:**
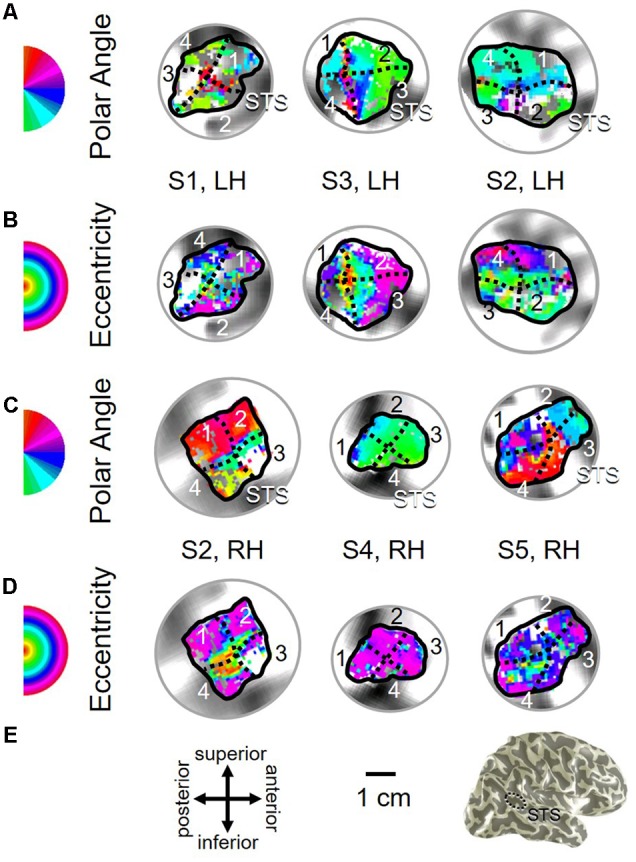
**Examples of the pSTS cluster across subjects. (A,B)** The pSTS cluster in left hemispheres (LH) from S1 and S3. **(C,D)** The pSTS cluster in right hemispheres (RH) from S2, S4, and S5. **(A,C)** pSTS polar angle representations on flattened representations of cortex. **(B,D)** pSTS eccentricity representations on the same flattened representations of cortex as pictured directly above in **(A,C)**. **(E)** *Left:* Approximate anatomical directions for **(A–D)**. Note that left and right hemispheres have been aligned to match in orientation. *Middle:* All flattened representations are set to the same scale; see 1 cm scale bar. *Right:* 3D right hemisphere denotes the cluster’s approximate location, which typically is along the posterior aspect of the superior temporal sulcus (STS; *dotted black line*). Other details area as in **Figure 2**.

Our fMRI measurements also revealed a new cluster of VFMs along the posterior aspect of the superior temporal sulcus (STS). This pSTS cluster contains four radially orthogonal hemifield VFMs, which we have termed pSTS-1, pSTS-2, pSTS-3, and pSTS-4, according to recent convention (**Figures 10, 11**) (Brewer et al., 2005; Kolster et al., 2010). The average coherence of each of these four VFMs was again clearly above our chosen conservative coherence threshold (co ≥ 0.25) and measured baseline noise (co ≤ 0.15) levels (**Figure 4**). The pSTS cluster tends to lie along the fundus and posterior bank of the superior portion of the STS and does not border any heretofore discovered VFM or cloverleaf cluster (**Figure 10C**). By convention, PSTS-1 is defined as the map with its posterior border lying in the middle of the posterior patch of lower vertical meridian representation (*cyan/blue*), bordering pSTS-4, and with its anterior border lying in the middle of the dorsal patch of upper vertical meridian representation (*magenta/red*), bordering pSTS-2. pSTS-2 is then the VFM anterior to pSTS-1 and dorsal to pSTS-3, which in turn borders pSTS-4 posteriorly. Compared to early and mid-level VFMs, the range of visual space representation (i.e., color span) was compressed, as expected for such small VFMs containing fewer voxels with relatively large pRFs; see below for further discussion. Even so, the boundary reversals between maps were clearly identifiable for all hemispheres in which we could define the pSTS cluster (9/10 hemispheres).

As illustrated in **Figure 5B**, the low-to-high vectors for each gradient were again reliably offset by roughly 90° in both VFMs, confirming that each VFM contains orthogonal eccentricity and polar-angle gradients. We were able to identify two orthogonal gradients to define all 4 VFMs in 9 hemispheres of our five subjects (e.g., 36 maps out of a possible 40); one subject (S5) lacked a clearly definable pSTS cluster in the left hemisphere. Out of the 36 measured maps, the majority spanned a full hemifield. For each individual VFM, 2–3 out of the 10 hemispheres had a compressed color range with a prominent representation of one quarterfield (e.g., only lower or upper visual field), but only a few voxels representing the other quarterfield (**Table 2**). As seen in the visual field coverage plots of **Figures 6C–F** and in the pRF size measurements of **Figures 8A,C**, all the pRFs within pSTS were close to the larger size seen in the peripheral regions in mid and early visual cortex, as expected for higher-order visual processing (Baizer et al., 1991; Ejima et al., 2003; Van Essen, 2003). The coverage plots further demonstrate the more peripheral pRF concentration in pSTS. There was still only a little overlap into the ipsilateral field along the vertical meridian especially near the fovea as measured with our stimuli; it is possible that some ipsilateral inputs also exist that were not strong enough compared to the contralateral inputs to be picked up by our measurements (Baizer et al., 1991; Ejima et al., 2003; Silver et al., 2005; Swisher et al., 2007; Silver and Kastner, 2009; Brewer and Barton, 2012b).

The surface area of the full pSTS cluster ranged across subjects from 388 mm^2^ to 764 mm^2^ (mean: 534 mm^2^; SEM: 66 mm^2^; **Figure 7A** and **Table 3**). This average cluster surface area is similar to what we measured for the V3A/B cluster, but note that the 4 VFMs in the pSTS cluster are then smaller than the 2 VFMs in the V3A/B cluster. The pSTS maps were very similar in size to each other within subjects, and the average sizes across subjects and hemispheres were comparable (**Figures 7C,D**), with an average area of 135 mm^2^ for pSTS-1 (SEM: 24 mm^2^), 147 mm^2^ for pSTS-2 (SEM: 21 mm^2^), 138 mm^2^ for pSTS-3 (SEM: 12 mm^2^), and 113 mm^2^ for pSTS-4 (SEM: 21 mm^2^). There were no significant differences in surface area between hemispheres for each VFM (**Figure 7F**, pSTS-1: *p* = 0.06; pSTS-2: *p* = 0.36; pSTS-3: *p* = 0.64; pSTS-4: *p* = 0.25).

For these slightly smaller pSTS VFMs of mean surface area of ∼133 mm^2^, we can approximate the mean VFM with a 6 × 6 matrix of 4.57 mm^2^ units with a total surface area of ∼165 mm^2^ (**Figure 1**). Therefore, the probability of observing either an eccentricity or polar-angle gradient in that cortical area is now (1/6)^(6^∗^6)^, or 9.70 ^∗^ 10^-29^. If we again triple the acceptable range of eccentricity values from 1/M to 3/M, the probability p of a gradient arising from noise keeping the other parameters the same becomes (3/6)^(6^∗^6)^, or 1.46 ^∗^ 10^-11^. For this smaller-sized VFM, the odds of two gradients arising in one hemisphere using the 3/M model are (1.46 ^∗^ 10^-11^)^2^, or 2.12 ^∗^ 10^-22^. This probability of a two-gradient map arising from noise is slightly higher than for V3A/B as expected for a smaller VFM composed of fewer voxels, but still very remote. In addition, we were able to measure complete pSTS clusters of 4 VFMs in 9 out of 10 hemispheres, as noted above (**Table 2**). The probability of measuring 2 gradients using the 3/M model in each of 9 hemispheres is (2.12 ^∗^ 10^-22^)^9^, or 8.56 ^∗^ 10^-196^. We thus reject the null hypothesis that the two gradients in each pSTS VFM observed in 9 hemispheres arose out of random noise.

To examine pSTS cluster pRF sizes and % surface area distributions, we again divided up the eccentricity representations into 10 eccentricity-band ROIs. We found that all 4 pSTS VFMs have pRF sizes that change little as a function of eccentricity (**Figures 8A,C**), and the surface area distribution is weighted heavily to the periphery, similar to the higher-order regions along the dorsal processing stream like the hMT+/TO cluster (**Figures 9A,C**) (Baizer et al., 1991; Brewer et al., 2002; Huk et al., 2002; Kolster et al., 2009, 2010; Brewer and Barton, 2012b). The average pRF radius for the cluster ranged from ∼4° to ∼6° across the measured eccentricity range (**Figure 8A**). Within the central 6°, the measurements of the individual VFMs had higher variability in pRF size than the outer range from 6 to 10° of eccentricity (**Figure 8C**). Note that there are no pRF centers that fall within the 0.5 eccentricity bin due to the relatively large pRF sizes within the pSTS cluster (Brewer and Barton, 2014, p. 212). In the more peripheral range, the 4 pSTS VFMs all had very similar pRF sizes of ∼4°. This increased foveal variability is not surprising, given the large shift in the % surface area to representations of the more peripheral regions (5–10°; **Figures 9A,C**); there were only a small number of foveal voxels within the central 6° from which to draw pRF size measurements for these VFMs. In addition, higher-order processing regions like we expect pSTS to be are often more significantly affected by alterations in attention. It is possible that the variability in pSTS foveal pRF sizes is due to different attentional or cognitive states among the subjects (Baizer et al., 1991; Silver et al., 2005; Silver and Kastner, 2009; Haak et al., 2012a); however, all subjects were successfully performing our visuospatial attentional task (Barton and Brewer, 2015). As in the V3A/B cluster, these measurements of a magnified peripheral representation are consistent with the expectations for expanded peripheral representations of visual space along the mid- and high-level regions of the dorsal stream (Baizer et al., 1991; Huk et al., 2002; Swisher et al., 2007; Kolster et al., 2009, 2010; Silver and Kastner, 2009).

### Clover Leaf Clusters as an Organizing Principle

**FIGURE 12:**
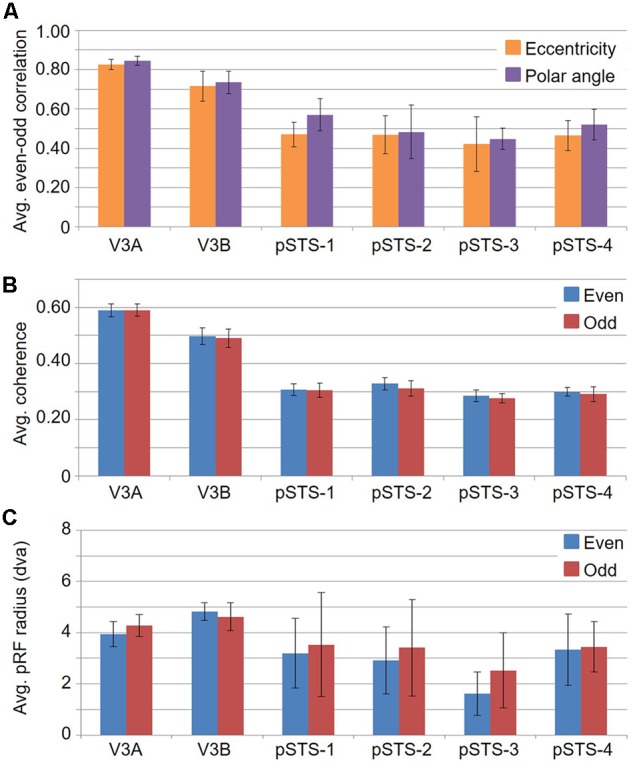
**Graphs of split-half even-odd reliability measurements. (A)** Even-odd correlation for pRF centers by eccentricity (orange) and polar angle (purple). **(B)** Coherence of even (blue) and odd (red) scans for each VFM. **(C)** Average pRF sizes for even (blue) and odd (red) scans in degrees of visual angle across the central 10° from fixation. All data are averaged across all 10 hemispheres. Error bars are SEM.

If cloverleaf clusters are fundamental to the human visual system, they should not only be seen throughout the visual hierarchy as we see here in mid- and higher-level visual cortex, but should also be reliably consistent and functionally differentiable (Wandell et al., 2005, 2007; Kolster et al., 2009, 2010; Barton et al., 2012; Brewer and Barton, 2012b). To characterize the reliability of these measurements, we performed an even-odd, split-half analysis of the functional scans for each subject (**Figure 12**; e.g., Swisher et al., 2007). First, note the relatively strong average even-odd correlations for eccentricity and polar angle in **Figure 12A**. The pRF model fits the pRF center in visual space to each voxel based on a 2D Gaussian pRF with a given size about the center. The average Pearson correlation coefficients of the eccentricity and polar angle pRF center fits for the V3A/B cluster are 0.77 (*p* < 0.01) and 0.79 (*p* < 0.01), respectively, and 0.46 (*p* < 0.01) and 0.51 (*p* < 0.01), respectively, for the pSTS cluster. Next, note the very similar average coherence across VFMs in **Figure 12B**. BOLD responses in these VFMs are not only well above threshold, but also consistent from scan to scan. Finally, note that the average pRF sizes for V3A and V3B are also very similar between the even and odd scans (**Figure 12C**). pSTS has slightly higher variability in the even-odd comparison of average pRF sizes, but this is not unexpected given the inclusion of the rather variable foveal measurements observed in each of the pSTS VFMs, as described above. Thus, each split-half measurement is remarkably reliable, especially when we consider the differing anatomical locations and functional characteristics of the VFMs presently evaluated (Callinan et al., 2003; Dougherty et al., 2003; Brewer and Barton, 2012b).

To examine whether the clusters are also functionally differentiable, first note that VFMs within a cluster have very similar total surface areas (**Figure 5**), and each cluster’s total surface area is consistent across subjects (**Table 3**). In addition, each cloverleaf cluster can be functionally differentiated by its pattern of coherence measurements (**Figure 4**), pRF sizes (**Figure 7**), and distribution of % surface area as a function of eccentricity (**Figure 9**). Note in particular that the distributions of pRF sizes have two patterns here: either the sizes remain roughly the same size across eccentricities as in the pSTS cluster, or they increase with more peripheral eccentricities as in the V3A/B cluster. Also note that there are two patterns of surface area distributions as eccentricities increase: the distribution gradually increases (V3A/B) or sharply increases (pSTS). These distinctions indicate that VFMs within individual cloverleaf clusters are not only anatomically, but also functionally related (Brewer et al., 2005; Wandell et al., 2005, 2007; Kolster et al., 2009, 2010; Moradi and Heeger, 2009; Brewer and Barton, 2012b).

## Discussion

Fundamentally, the VFMs presently identified indicate the presence of visuospatial organization in the mid and high tiers of the cortical visual processing hierarchy. We provide evidence for four novel VFMs organized into a cloverleaf cluster in the pSTS and novel pRF-based analyses of the previously identified, mid-level VFMs V3A and V3B, which together form the V3A/B cloverleaf cluster. These clusters and the VFMs within have consistent anatomical locations (**Figures 2, 3, 10, 11** and **Tables 1, 2**), coherence (**Figure 4**), surface areas (**Figure 7** and **Table 3**), pRF sizes (**Figure 8**), and distributions of pRF centers across surface area (**Figures 6, 9**). In addition, split-half analyses reveal that each of these measurements is highly reliable (**Figure 12**). Finally, the individual cloverleaf clusters are differentiable by differences in these measurements.

The present results add significant weight to the theory that a unifying matrix of visuospatial organization is maintained in VFMs throughout the visual hierarchy, despite the diverse computations being performed across regions (e.g., Van Essen, 2003; Wandell et al., 2007). In low-level VFMs, precise measurements are taken of low-level visual features in a particular retinal location, which are built up into more complicated localized representations as they are processed through the cortical hierarchy. Despite having large receptive fields, higher-order visual cortex may still maintain visuospatial organization by maintaining just enough dispersion of pRF centers to allow for slightly different preferred tuning of responses to visual space (Lehky and Sereno, 2011). The presence of organized representations of visual space in high-order regions can still allow for the stimulus size and position invariances frequently described across higher-order object- and face-responsive visual regions, as such invariance can arise in regions simply with very large receptive fields (Dumoulin and Wandell, 2008; Brewer and Barton, 2012b; Haak et al., 2012b). It is possible that the majority of higher-order visual areas are organized according to visual space, maintaining retinotopically organized, dispersed receptive field centers despite increasingly large receptive field sizes (Hagler et al., 2007; Sereno and Lehky, 2011; Lehky et al., 2015).

Whether the spatial organization remains truly retinotopic or changes to a broader spatiotopic organization is still under investigation and can’t be determined with the visual field mapping methods used here (Sereno et al., 2001; Hagler et al., 2007). In either case, such widespread maintenance of visuospatial organization allows for a common reference frame through which information can be passed up or down the visual hierarchy. Theories of attention in which higher-order visual-attentional areas are able to affect many lower-level visual areas simultaneously in spatially specific patterns can be explained through the use of such visual-location-based “channels” (e.g., Sereno et al., 2001; Silver et al., 2005; Sereno and Amador, 2006; Silver and Kastner, 2009). In some cases, it is also possible that visuospatial organization is maintained despite visual location information not being critical to the computations of that specific area simply because it would be too disruptive or costly during development to change the organization once it has been established at the level of the retina or earlier visual cortex.

The present work does not address functional localization of the particular types of processing occurring in either cloverleaf cluster, which we expect will be a fruitful line of future research. Also of interest is the homology of cloverleaf cluster organization of human and non-human primates. Previous research in V3A has demonstrated that it plays a role in human motion processing, and its retinotopic characteristics are similar between humans and macaque (Tootell et al., 1997; Brewer et al., 2002). The functional characteristics of V3B have not been fully worked out. Prior related measurements as well as cloverleaf cluster organization predictions suggest that its functional characteristics may be similar to V3A (Tootell et al., 1997; Smith et al., 1998, 2001; Press et al., 2001). As for the pSTS cluster, the general region in which these VFMs lie has been implicated in high-order visual processing dealing with complex aspects of face and motion processing (Hoffman and Haxby, 2000; Grossman and Blake, 2002). In addition, this cortex has been associated with high-order multisensory processing, including the integration of auditory and visual information about objects (Beauchamp et al., 2004). The pSTS VFMs do not yet have a clear homology to similar visuospatial organization in macaque. Additional functional measurements may help clarify whether such a homology exists; with 25 million years of divergent evolution between the species, the pSTS cluster may be in a region of high-order visual processing unique to humans (Hedges and Kumar, 2003).

We expect that cloverleaf clusters will be found to be a fundamental organizational unit for VFMs across visual cortex (Brewer et al., 2005; Wandell et al., 2005; Kolster et al., 2009, 2010). Not only does the cloverleaf cluster appear to be an efficient way to group neurons performing related computations, but one can also imagine that a duplication of an organizational unit such as the cloverleaf cluster during evolution could facilitate the development of expanded or even novel visual computations for an emerging species (Krubitzer, 2007; Brewer and Barton, 2016). Furthermore, we have speculated for some time that the cloverleaf cluster organizational pattern may extend to other types of sensory cortex. Once a particular organizational unit such as the cloverleaf cluster has arisen in one sensory modality, the same organizational unit might be duplicated and repeated across the brain as it evolved, following consistent genetic mechanisms during development (Krubitzer, 2007). As research of topographical representations expands in other sensory domains (e.g., audition, somatosensation), we could then use the predictions of CFM and cloverleaf cluster organization seen in vision to guide measurements of similar topographic groupings in these regions (Sanchez-Panchuelo et al., 2010; Barton et al., 2012; Mancini et al., 2012). Evidence supporting this assertion comes from the human auditory system, where the present authors recently collaborated to measure the first auditory field maps (AFMs) along Heschl’s gyrus (HG; Barton et al., 2012; Brewer and Barton, 2016). Human AFMs exhibit very similar organization to the presently measured VFMs: they each consist of two orthogonal sensory dimensions, and six of them are organized into the first measured radially orthogonal auditory cloverleaf cluster, the HG cluster. Taken together with our present results in mid- and higher-order visual cortex, these findings suggest that the cloverleaf cluster macrostructural organization is indeed fundamental to such sensory systems, providing a basic framework for the complex processing and analysis of input from sensory receptors.

## Author Contrbutions

BB and AB conceived of and conducted the experiments. BB and AB processed and analyzed the data. AB and BB wrote and revised the manuscript.

## Conflict of Interest Statement

The authors declare that the research was conducted in the absence of any commercial or financial relationships that could be construed as a potential conflict of interest.

## Footnotes

1 https://www.fmrib.ox.ac.uk/fsl

2 http://white.stanford.edu/software/

## References

[B1] Amano K. Wandell B. A. Dumoulin S. O. (2009). Visual field maps, population receptive field sizes, and visual field coverage in the human MT+ complex. *J. Neurophysiol.* 102 2704–2718. 10.1152/jn.00102.2009 19587323 PMC2777836

[B2] Arcaro M. J. McMains S. A. Singer B. D. Kastner S. (2009). Retinotopic organization of human ventral visual cortex. *J. Neurosci.* 29 10638–10652. 10.1523/JNEUROSCI.2807-09.2009 19710316 PMC2775458

[B3] Baizer J. S. Ungerleider L. G. Desimone R. (1991). Organization of visual inputs to the inferior temporal and posterior parietal cortex in macaques. *J. Neurosci.* 11 168–190. 1702462 10.1523/JNEUROSCI.11-01-00168.1991 PMC6575184

[B4] Bartels A. Zeki S. (2000). The architecture of the colour centre in the human visual brain: new results and a review. *Eur. J. Neurosci.* 12 172–193. 10651872 10.1046/j.1460-9568.2000.00905.x

[B5] Barton B. Brewer A. A. (2015). fMRI of the rod scotoma elucidates cortical rod pathways and implications for lesion measurements. *Proc. Natl. Acad. Sci. U.S.A.* 112 5201–5206. 10.1073/pnas.1423673112 25848028 PMC4413354

[B6] Barton B. Venezia J. H. Saberi K. Hickok G. Brewer A. A. (2012). Orthogonal acoustic dimensions define auditory field maps in human cortex. *Proc. Natl. Acad. Sci. U.S.A.* 109 20738–20743. 10.1073/pnas.1213381109 23188798 PMC3528571

[B7] Baseler H. A. Brewer A. A. Sharpe L. T. Morland A. B. Jagle H. Wandell B. A. (2002). Reorganization of human cortical maps caused by inherited photoreceptor abnormalities. *Nat. Neurosci.* 5 364–370. 10.1038/nn817 11914722

[B8] Baseler H. A. Gouws A. Haak K. V. Racey C. Crossland M. D. Tufail A. (2011). Large-scale remapping of visual cortex is absent in adult humans with macular degeneration. *Nat. Neurosci.* 14 649–655. 10.1038/nn.2793 21441924

[B9] Baseler H. A. Morland A. B. Wandell B. A. (1999). Topographic organization of human visual areas in the absence of input from primary cortex. *J. Neurosci.* 19 2619–2627. 10087075 10.1523/JNEUROSCI.19-07-02619.1999 PMC6786078

[B10] Beauchamp M. S. Lee K. E. Argall B. D. Martin A. (2004). Integration of auditory and visual information about objects in superior temporal sulcus. *Neuron* 41 809–823. 10.1016/S0896-6273(04)00070-4 15003179

[B11] Boynton G. M. Engel S. A. Glover G. H. Heeger D. J. (1996). Linear systems analysis of functional magnetic resonance imaging in human V1. *J. Neurosci.* 16 4207–4221. 8753882 10.1523/JNEUROSCI.16-13-04207.1996 PMC6579007

[B12] Brainard D. H. (1997). The psychophysics toolbox. *Spat. Vis.* 10 433–436. 9176952

[B13] Brewer A. A. (2009). Visual maps: to merge or not to merge. *Curr. Biol.* 19 R945–R947. 10.1016/j.cub.2009.09.016 19889370

[B14] Brewer A. A. Barton B. (2012a). Effects of healthy aging on human primary visual cortex. *Health* 4 695–702. 10.4236/health.2012.429109 26881051 PMC4752674

[B15] Brewer A. A. Barton B. (2012b). “Visual field map organization in human visual cortex,” in *Visual Cortex - Current Status and Perspectives* eds Molotchnikoff S. Rouat J. (Rijeka: InTech) 29–60.

[B16] Brewer A. A. Barton B. (2014). Visual cortex in aging and Alzheimer’s disease: changes in visual field maps and population receptive fields. *Front. Psychol.* 5:74 10.3389/fpsyg.2014.00074 PMC3916727 24570669

[B17] Brewer A. A. Barton B. (2016). Maps of the auditory cortex. *Annu. Rev. Neurosci.* 39 385–407. 10.1146/annurev-neuro-070815-014045 27145914 PMC6436392

[B18] Brewer A. A. Liu J. Wade A. R. Wandell B. A. (2005). Visual field maps and stimulus selectivity in human ventral occipital cortex. *Nat. Neurosci.* 8 1102–1109. 10.1038/nn1507 16025108

[B19] Brewer A. A. Press W. A. Logothetis N. K. Wandell B. A. (2002). Visual areas in macaque cortex measured using functional magnetic resonance imaging. *J. Neurosci.* 22 10416–10426. 12451141 10.1523/JNEUROSCI.22-23-10416.2002 PMC6758759

[B20] Callinan P. A. Hedges D. J. Salem A. H. Xing J. Walker J. A. Garber R. K. (2003). Comprehensive analysis of Alu-associated diversity on the human sex chromosomes. *Gene* 317 103–110. 10.1016/S0378-1119(03)00662-0 14604797

[B21] Chklovskii D. B. Koulakov A. A. (2004). Maps in the brain: what can we learn from them? *Annu. Rev. Neurosci.* 27 369–392. 10.1146/annurev.neuro.27.070203.144226 15217337

[B22] DeYoe E. A. Carman G. J. Bandettini P. Glickman S. Wieser J. Cox R. (1996). Mapping striate and extrastriate visual areas in human cerebral cortex. *Proc. Natl. Acad. Sci. U.S.A.* 93 2382–2386. 8637882 10.1073/pnas.93.6.2382 PMC39805

[B23] Dougherty R. F. Koch V. M. Brewer A. A. Fischer B. Modersitzki J. Wandell B. A. (2003). Visual field representations and locations of visual areas V1/2/3 in human visual cortex. *J. Vis.* 3 586–598. 10.1167/3.10.1 14640882

[B24] Duffy K. R. Hubel D. H. (2007). Receptive field properties of neurons in the primary visual cortex under photopic and scotopic lighting conditions. *Vision Res.* 47 2569–2574. 10.1016/j.visres.2007.06.009 17688906 PMC2951600

[B25] Dumoulin S. O. Wandell B. A. (2008). Population receptive field estimates in human visual cortex. *Neuroimage* 39 647–660. 10.1016/j.neuroimage.2007.09.034 17977024 PMC3073038

[B26] Ejima Y. Takahashi S. Yamamoto H. Fukunaga M. Tanaka C. Ebisu T. (2003). Interindividual and interspecies variations of the extrastriate visual cortex. *Neuroreport* 14 1579–1583. 10.1097/01.wnr.0000086098.47480.44 14502080

[B27] Engel S. A. Glover G. H. Wandell B. A. (1997). Retinotopic organization in human visual cortex and the spatial precision of functional MRI. *Cereb. Cortex* 7 181–192. 10.1093/cercor/7.2.181 9087826

[B28] Engel S. A. Rumelhart D. E. Wandell B. A. Lee A. T. Glover G. H. Chichilnisky E. J. (1994). fMRI of human visual cortex. *Nature* 369 525 10.1038/369525a0 8031403

[B29] Fine I. Wade A. R. Brewer A. A. May M. G. Goodman D. F. Boynton G. M. (2003). Long-term deprivation affects visual perception and cortex. *Nat. Neurosci.* 6 915–916. 10.1038/nn1102 12937420

[B30] Friston K. J. Fletcher P. Josephs O. Holmes A. Rugg M. D. Turner R. (1998). Event-related fMRI: characterizing differential responses. *Neuroimage* 7 30–40. 10.1006/nimg.1997.0306 9500830

[B31] Grill-Spector K. Malach R. (2004). The human visual cortex. *Annu. Rev. Neurosci.* 27 649–677. 15217346 10.1146/annurev.neuro.27.070203.144220

[B32] Grossman E. D. Blake R. (2002). Brain areas active during visual perception of biological motion. *Neuron* 35 1167–1175. 10.1016/S0896-6273(02)00897-8 12354405

[B33] Haak K. V. Cornelissen F. W. Morland A. B. (2012a). Population receptive field dynamics in human visual cortex. *PLoS ONE* 7:e37686 10.1371/journal.pone.0037686 PMC3359387 22649551

[B34] Haak K. V. Winawer J. Harvey B. M. Renken R. Dumoulin S. O. Wandell B. A. (2012b). Connective field modeling. *Neuroimage* 66C 376–384. 10.1016/j.neuroimage.2012.10.037 PMC3769486 23110879

[B35] Hadjikhani N. Tootell R. B. (2000). Projection of rods and cones within human visual cortex. *Hum. Brain Mapp.* 9 55–63. 10643730 10.1002/(SICI)1097-0193(2000)9:1<55::AID-HBM6>3.0.CO;2-U PMC6871842

[B36] Hagler D. J. Jr. Riecke L. Sereno M. I. (2007). Parietal and superior frontal visuospatial maps activated by pointing and saccades. *Neuroimage* 35 1562–1577. 10.1016/j.neuroimage.2007.01.033 17376706 PMC2752728

[B37] Hasson U. Levy I. Behrmann M. Hendler T. Malach R. (2002). Eccentricity bias as an organizing principle for human high-order object areas. *Neuron* 34 479–490. 11988177 10.1016/s0896-6273(02)00662-1

[B38] Hedges S. B. Kumar S. (2003). Genomic clocks and evolutionary timescales. *Trends Genet.* 19 200–206. 10.1016/S0168-9525(03)00053-2 12683973

[B39] Hoffman E. A. Haxby J. V. (2000). Distinct representations of eye gaze and identity in the distributed human neural system for face perception. *Nat. Neurosci.* 3 80–84. 10.1038/71152 10607399

[B40] Hoffmann M. B. Kaule F. R. Levin N. Masuda Y. Kumar A. Gottlob I. (2012). Plasticity and stability of the visual system in human achiasma. *Neuron* 75 393–401. 10.1016/j.neuron.2012.05.026 22884323 PMC3427398

[B41] Hoffmann M. B. Thieme H. Liedecke K. Meltendorf S. Zenker M. Wieland I. (2015). Visual pathways in humans with ephrin-B1 deficiency associated with the cranio-fronto-nasal syndrome. *Invest. Ophthalmol. Vis. Sci.* 56 7427–7437. 10.1167/iovs.15-17705 26580852

[B42] Hoffmann M. B. Tolhurst D. J. Moore A. T. Morland A. B. (2003). Organization of the visual cortex in human albinism. *J. Neurosci.* 23 8921–8930. 14523094 10.1523/JNEUROSCI.23-26-08921.2003 PMC6740392

[B43] Hubel D. H. Howe P. D. Duffy A. M. Hernandez A. (2009). Scotopic foveal afterimages. *Perception* 38 313–316. 19400439 10.1068/p6103

[B44] Huk A. C. Dougherty R. F. Heeger D. J. (2002). Retinotopy and functional subdivision of human areas MT and MST. *J. Neurosci.* 22 7195–7205. 12177214 10.1523/JNEUROSCI.22-16-07195.2002 PMC6757870

[B45] Kaas J. (1997). “Theories of visual cortex organisation in primates,” in *Cerebral Cortex: Extrastriate Cortex in Primates* eds Rockland K. S. Kass J. H. Peters A. (New York, NY: Plenum Press) 91–125.

[B46] Kaas J. H. (1997). Topographic maps are fundamental to sensory processing. *Brain Res. Bull.* 44 107–112. 9292198 10.1016/s0361-9230(97)00094-4

[B47] Kastner S. De Weerd P. Pinsk M. A. Elizondo M. I. Desimone R. Ungerleider L. G. (2001). Modulation of sensory suppression: implications for receptive field sizes in the human visual cortex. *J. Neurophysiol.* 86 1398–1411. 11535686 10.1152/jn.2001.86.3.1398

[B48] Kolster H. Mandeville J. B. Arsenault J. T. Ekstrom L. B. Wald L. L. Vanduffel W. (2009). Visual field map clusters in macaque extrastriate visual cortex. *J. Neurosci.* 29 7031–7039. 10.1523/JNEUROSCI.0518-09.2009 19474330 PMC2749229

[B49] Kolster H. Peeters R. Orban G. A. (2010). The retinotopic organization of the human middle temporal area MT/V5 and its cortical neighbors. *J. Neurosci.* 30 9801–9820. 10.1523/JNEUROSCI.2069-10.2010 20660263 PMC6632824

[B50] Krubitzer L. (2007). The magnificent compromise: cortical field evolution in mammals. *Neuron* 56 201–208. 10.1016/j.neuron.2007.10.002 17964240

[B51] Larsson J. Heeger D. J. (2006). Two retinotopic visual areas in human lateral occipital cortex. *J. Neurosci.* 26 13128–13142. 10.1523/JNEUROSCI.1657-06.2006 17182764 PMC1904390

[B52] Lehky S. R. Sereno A. B. (2011). Population coding of visual space: modeling. *Front. Comput. Neurosci.* 4:155 10.3389/fncom.2010.00155 PMC3034232 21344012

[B53] Lehky S. R. Sereno M. E. Sereno A. B. (2015). Characteristics of eye-position gain field populations determine geometry of visual space. *Front. Integr. Neurosci.* 9:72 10.3389/fnint.2015.00072 PMC4718998 26834587

[B54] Maes F. Collignon A. Vandermeulen D. Marchal G. Suetens P. (1997). Multimodality image registration by maximization of mutual information. *IEEE Trans. Med. Imaging* 16 187–198. 10.1109/42.563664 9101328

[B55] Malach R. Levy I. Hasson U. (2002). The topography of high-order human object areas. *Trends Cogn. Sci.* 6 176–184. 11912041 10.1016/s1364-6613(02)01870-3

[B56] Mancini F. Haggard P. Iannetti G. D. Longo M. R. Sereno M. I. (2012). Fine-grained nociceptive maps in primary somatosensory cortex. *J. Neurosci.* 32 17155–17162. 10.1523/JNEUROSCI.3059-12.2012 23197708 PMC3529201

[B57] Mitchison G. (1991). Neuronal branching patterns and the economy of cortical wiring. *Proc. Biol. Sci.* 245 151–158. 10.1098/rspb.1991.0102 1682939

[B58] Moradi F. Heeger D. J. (2009). Inter-ocular contrast normalization in human visual cortex. *J. Vis.* 9 13 11–22. 10.1167/9.3.13 PMC2747122 19757952

[B59] Morland A. B. Baseler H. A. Hoffmann M. B. Sharpe L. T. Wandell B. A. (2001). Abnormal retinotopic representations in human visual cortex revealed by fMRI. *Acta Psychol.* 107 229–247. 10.1016/s0001-6918(01)00025-7 11388137

[B60] Muckli L. Naumer M. J. Singer W. (2009). Bilateral visual field maps in a patient with only one hemisphere. *Proc. Natl. Acad. Sci. U.S.A.* 106 13034–13039. 10.1073/pnas.0809688106 19620732 PMC2713389

[B61] Nestares O. Heeger D. J. (2000). Robust multiresolution alignment of MRI brain volumes. *Magn. Reson. Med.* 43 705–715. 10800036 10.1002/(sici)1522-2594(200005)43:5<705::aid-mrm13>3.0.co;2-r

[B62] Op de Beeck H. P. Haushofer J. Kanwisher N. G. (2008). Interpreting fMRI data: maps, modules and dimensions. *Nat. Rev. Neurosci.* 9 123–135. 10.1038/nrn2314 18200027 PMC2731480

[B63] Pelli D. G. (1997). The VideoToolbox software for visual psychophysics: transforming numbers into movies. *Spat. Vis.* 10 437–442. 9176953

[B64] Press W. A. Brewer A. A. Dougherty R. F. Wade A. R. Wandell B. A. (2001). Visual areas and spatial summation in human visual cortex. *Vision Res.* 41 1321–1332. 11322977 10.1016/s0042-6989(01)00074-8

[B65] Sanchez-Panchuelo R. M. Francis S. Bowtell R. Schluppeck D. (2010). Mapping human somatosensory cortex in individual subjects with 7T functional MRI. *J. Neurophysiol.* 103 2544–2556. 10.1152/jn.01017.2009 20164393 PMC2867563

[B66] Sereno A. B. Amador S. C. (2006). Attention and memory-related responses of neurons in the lateral intraparietal area during spatial and shape-delayed match-to-sample tasks. *J. Neurophysiol.* 95 1078–1098. 10.1152/jn.00431.2005 16221750

[B67] Sereno A. B. Lehky S. R. (2011). Population coding of visual space: comparison of spatial representations in dorsal and ventral pathways. *Front. Comput. Neurosci.* 4:159 10.3389/fncom.2010.00159 PMC3034230 21344010

[B68] Sereno M. I. Dale A. M. Reppas J. B. Kwong K. K. Belliveau J. W. Brady T. J. (1995a). Borders of multiple human visual areas in humans revealed by functional MRI. *Science* 268 889–893. 7754376 10.1126/science.7754376

[B69] Sereno M. I. Dale A. M. Reppas J. B. Kwong K. K. Belliveau J. W. Brady T. J. (1995b). Borders of multiple visual areas in humans revealed by functional magnetic resonance imaging. *Science* 268 889–893. 7754376 10.1126/science.7754376

[B70] Sereno M. I. McDonald C. T. Allman J. M. (1994). Analysis of retinotopic maps in extrastriate cortex. *Cereb. Cortex* 6 601–620. 10.1093/cercor/4.6.601 7703687

[B71] Sereno M. I. Pitzalis S. Martinez A. (2001). Mapping of contralateral space in retinotopic coordinates by a parietal cortical area in humans. *Science* 294 1350–1354. 10.1126/science.1063695 11701930

[B72] Shapley R. Hawken M. Xing D. (2007). The dynamics of visual responses in the primary visual cortex. *Prog. Brain Res.* 165 21–32. 10.1016/S0079-6123(06)65003-6 17925238

[B73] Shipp S. Watson J. D. Frackowiak R. S. Zeki S. (1995). Retinotopic maps in human prestriate visual cortex: the demarcation of areas V2 and V3. *Neuroimage* 2 125–132. 10.1006/nimg.1995.1015 9343595

[B74] Silver M. A. Kastner S. (2009). Topographic maps in human frontal and parietal cortex. *Trends Cogn. Sci.* 13 488–495. 10.1016/j.tics.2009.08.005 19758835 PMC2767426

[B75] Silver M. A. Ress D. Heeger D. J. (2005). Topographic maps of visual spatial attention in human parietal cortex. *J. Neurophysiol.* 94 1358–1371. 10.1152/jn.01316.2004 15817643 PMC2367310

[B76] Smirnakis S. M. Brewer A. A. Schmid M. C. Tolias A. S. Schuz A. Augath M. (2005). Lack of long-term cortical reorganization after macaque retinal lesions. *Nature* 435 300–307. 10.1038/nature03495 15902248

[B77] Smith A. T. Greenlee M. W. Singh K. D. Kraemer F. M. Hennig J. (1998). The processing of first- and second-order motion in human visual cortex assessed by functional magnetic resonance imaging (fMRI). *J. Neurosci.* 18 3816–3830. 9570811 10.1523/JNEUROSCI.18-10-03816.1998 PMC6793149

[B78] Smith A. T. Singh K. D. Williams A. L. Greenlee M. W. (2001). Estimating receptive field size from fMRI data in human striate and extrastriate visual cortex. *Cereb. Cortex* 11 1182–1190. 11709489 10.1093/cercor/11.12.1182

[B79] Swisher J. D. Halko M. A. Merabet L. B. McMains S. A. Somers D. C. (2007). Visual topography of human intraparietal sulcus. *J. Neurosci.* 27 5326–5337. 10.1523/JNEUROSCI.0991-07.2007 17507555 PMC6672354

[B80] Teo P. C. Sapiro G. Wandell B. A. (1997). Creating connected representations of cortical gray matter for functional MRI visualization. *IEEE Trans. Med. Imaging* 16 852–863. 10.1109/42.650881 9533585

[B81] Tootell R. B. Hadjikhani N. Hall E. K. Marrett S. Vanduffel W. Vaughan J. T. (1998). The retinotopy of visual spatial attention. *Neuron* 21 1409–1422. 10.1016/S0896-6273(00)80659-5 9883733

[B82] Tootell R. B. Mendola J. D. Hadjikhani N. K. Ledden P. J. Liu A. K. Reppas J. B. (1997). Functional analysis of V3A and related areas in human visual cortex. *J. Neurosci.* 17 7060–7078. 9278542 10.1523/JNEUROSCI.17-18-07060.1997 PMC6573277

[B83] Tootell R. B. Reppas J. B. Kwong K. K. Malach R. Born R. T. Brady T. J. (1995). Functional analysis of human MT and related visual cortical areas using magnetic resonance imaging. *J. Neurosci.* 15 3215–3230. 7722658 10.1523/JNEUROSCI.15-04-03215.1995 PMC6577785

[B84] Tyler C. W. Wade A. R. (2005). Extended concepts of occipital retinotopy. *Curr. Med. Imaging Rev.* 1 319–329.

[B85] Van Essen D. C. (2003). *Organization of Visual Areas in Macaque and Human Cerebral Cortex.* Boston, MA: Bradford.

[B86] Wandell B. A. Brewer A. A. Dougherty R. F. (2005). Visual field map clusters in human cortex. *Philos. Trans. R. Soc. Lond. B Biol. Sci.* 360 693–707. 10.1098/rstb.2005.1628 15937008 PMC1569486

[B87] Wandell B. A. Chial S. Backus B. T. (2000). Visualization and measurement of the cortical surface. *J. Cogn. Neurosci.* 12 739–752. 11054917 10.1162/089892900562561

[B88] Wandell B. A. Dumoulin S. O. Brewer A. A. (2007). Visual field maps in human cortex. *Neuron* 56 366–383. 10.1016/j.neuron.2007.10.012 17964252

